# Unusual neutrophilic morphology in influenza-A-induced septic shock: Blue-green inclusions and phagocytosis of autologous cells in a toddler

**DOI:** 10.3389/fcimb.2025.1684054

**Published:** 2025-11-01

**Authors:** Xiaomei Wang, Xiang Ma, Ye Zeng, Changzhen Li

**Affiliations:** Department of Laboratory Medicine, Wuhan Children’s Hospital (Wuhan Maternal and Child Healthcare Hospital), Tongji Medical College, Huazhong University of Science & Technology, Wuhan, China

**Keywords:** influenza A virus, pediatric, septic shock, blue-green inclusions, neutrophilic hemophagocytosis

## Abstract

*Influenza A virus* is a major cause of seasonal respiratory illness, typically self-limiting in children. However, in rare instances, it can lead to fulminant systemic complications such as acute necrotizing encephalopathy (ANE), sepsis-induced shock, and multiple organ dysfunction syndrome (MODS). We report a rare case of a 2-year-3-month-old girl who developed sepsis-induced shock secondary to influenza A infection. The patient presented with sudden high fever, frequent seizures, impaired consciousness, and respiratory failure. Rapid clinical deterioration ensued, with evidence of profound hyperinflammatory response, coagulopathy, hepatic and myocardial injury, and lactic acidosis. Neurophysiological findings were consistent with ANE. Notably, peripheral blood smear revealed two rare hematologic features: blue-green neutrophilic inclusions (BGNIs) and neutrophilic phagocytosis of both lymphocytes and erythrocytes. These findings—unprecedented in the context of pediatric influenza A infection—may reflect severe immune dysregulation and oxidative stress. Despite aggressive multidisciplinary interventions, the patient succumbed to MODS and disseminated intravascular coagulation (DIC) on day 4 of illness. This is the first reported pediatric case of influenza A infection with concurrent BGNIs and neutrophilic hemophagocytosis in peripheral blood. These morphological abnormalities may serve as hematological markers of life-threatening systemic inflammation in viral sepsis. Awareness and prompt recognition of these features may aid in prognostication and clinical decision making for critically ill children with influenza.

## Introduction

*Influenza A virus* is a major etiological agent of seasonal respiratory infections worldwide, with young children, the elderly, and immunocompromised individuals being particularly susceptible. Although the majority of pediatric cases are self-limiting, a subset of patients may develop severe and potentially fatal complications, including pneumonia, acute respiratory distress syndrome (ARDS), and sepsis-induced shock (Kalil and Thomas, 2019). Sepsis-induced shock represents a critical condition characterized by a dysregulated host immune response to infection, resulting in circulatory failure and progressive multi-organ dysfunction (Singer et al., 2016). In pediatric populations—especially among infants and toddlers—the clinical manifestations of sepsis may be subtle or atypical, thereby posing significant diagnostic challenges.

In the context of severe infection, a rare but noteworthy hematological finding is the presence of blue-green cytoplasmic inclusions within neutrophils. These blue-green neutrophilic inclusions (BGNIs), also referred to as “death bodies,” are bright green structures observed in the cytoplasm of neutrophils or monocytes and have been closely associated with critical illnesses such as acute liver failure and lactic acidosis (Hodgson et al., 2015). These distinctive inclusions have been reported across a wide range of infectious diseases, including bacterial infections (e.g., *Escherichia coli (* Jazaerly and Gabali, 2014), *Pandoraea* spp (Zhang et al., 2023), *Neisseria meningitidis (* Shackleton et al., 2021)), viral infections (e.g., SARS-CoV-2 (Cantu et al., 2020), severe fever with *Thrombocytopenia Syndrome Virus* [SFTSV] (Jiang et al., 2023), *Yellow Fever virus (* Martins et al., 2022)), fungal infections (e.g., *Aspergillus* spp), and parasitic infections such as malaria (Merino et al., 2021). However, their association with *influenza A virus* infection remains exceptionally rare.

Herein, we present a rare case of a 2-year-3-month-old girl who developed sepsis-induced shock secondary to *influenza A virus* infection. Peripheral blood smear examination revealed the presence of blue-green inclusions in neutrophils, accompanied by neutrophilic phagocytosis of both lymphocytes and erythrocytes. To our knowledge, this is the first report describing the simultaneous occurrence of these two rare morphological changes in a pediatric patient following influenza A infection. The clinical course and management of this patient are detailed below.

## Case presentation

A 2-year-3-month-old girl, born full term via cesarean section through *in vitro* fertilization, presented with an unremarkable medical history except for a diagnosis of patent ductus arteriosus during infancy, for which no intervention was performed. She had no history of trauma or drug allergies.

On December 9, 2024, she developed a sudden onset of high fever, peaking at 40.5°C, accompanied by chills, rigors, and altered consciousness. Four seizure episodes occurred on the same day, each characterized by upward gaze and limb convulsions. During some episodes, the patient exhibited visual hallucinations, reportedly “seeing wolves.” Postictal recovery of consciousness was incomplete between episodes. At the outpatient clinic, a rapid antigen test was performed on a nasopharyngeal swab using a colloidal gold immunochromatographic assay (BioPerfectus, Taizhou, China), which was positive for *influenza A virus* antigen.

Following the fourth seizure, she developed acute respiratory distress, perioral cyanosis, and unresponsiveness to external stimuli. There was no vomiting, and caregivers denied any history of foreign body aspiration. At 00:25 on December 10, 2024, she was urgently admitted to the emergency department of our hospital. Physical examination revealed somnolence, positive signs of respiratory distress (intercostal, suprasternal, and subcostal retractions), and prolonged capillary refill time (3–4 seconds). Immediate interventions included endotracheal intubation, gastric decompression, and airway suctioning, which revealed large amounts of gastric content. Based on clinical presentation and initial investigations, she was admitted to the Pediatric Intensive Care Unit (PICU) with presumptive diagnoses of central nervous system infection, respiratory failure, influenza A infection, and aspiration pneumonia.

## Laboratory and imaging findings

On admission (December 10, 2024), nucleic acid testing was performed using a multiplex respiratory pathogen detection kit (Ningbo Health Gene Technologies, China), which employs RT-PCR combined with capillary electrophoresis for endpoint analysis. The panel included *influenza A virus* (H7N9, H1N1, H3N2, H5N2, and H1N1 2009), seasonal H3N2, *influenza B virus* (Victoria and Yamagata lineages), *adenovirus* (types B, C, and E), *bocavirus*, *rhinovirus*, *parainfluenza virus* (types 1–4), *coronaviruses* (229E, OC43, NL63, HKU1), *respiratory syncytial virus* (groups A and B), *metapneumovirus*, and atypical bacteria (*Mycoplasma pneumoniae* and *Chlamydia* sp*ecies*, including *C. trachomatis* and *C. pneumoniae*). The results confirmed influenza A H1N1 (2009) RNA positivity, based on the simultaneous detection of both InfA and 09H1 specific peaks, while all other tested pathogens were negative.

Inflammatory markers were markedly elevated, with interleukin-6 (IL-6) reaching 32,367.87 pg/ml and interleukin-10 (IL-10) at 354.37 pg/ml. Both high-sensitivity C-reactive protein (hsCRP) and procalcitonin (PCT) levels were increased. Serum ferritin level was profoundly elevated to 20,269.98 ng/ml. Arterial blood gas analysis revealed metabolic acidosis. Liver function tests showed markedly elevated alanine aminotransferase (ALT) and aspartate aminotransferase (AST). Renal function was impaired, with increased levels of blood urea nitrogen (BUN) and creatinine. Cardiac enzyme analysis demonstrated remarkably elevated lactate dehydrogenase (LDH). Additionally, coagulopathy with electrolyte disturbances was noted (details in **Table 1**).

**Table 1:** Abnormal results of routine laboratory examination of the patient on admission (color code: red = elevated, blue = decreased).

Parameter (unit)	Results	Reference intervals
Complete blood count and leukocyte differential count tests		
Percentage of lymphocyte (%)	73.3	23–69
Neutrophil count (x10^9^/L)	0.96	1.2–7
Percentage of neutrophil (%)	17.9	22–65
Erythrocyte count (x1012/L)	3.37	4.0–5.5
Hemoglobin (g/L)	99	112–149
Hematocrit (%)	30	34–43
Platelet count (x10^9^/L)	129	188–472
Routine chemical testing		
Total protein (g/L)	56.4	61–79
Aspartate aminotransferase (U/L)	388.0	14–44
Alanine aminotransferase (U/L)	132.0	7–30
Alkaline phosphatase (U/L)	461.0	143–406
Blood urea nitrogen (mmol/L)	8.3	2.5–6.5
Creatinine (µmol/L)	80.3	19–44
Uric acid (µmol/L)	459.0	208–428
Cystatin C (mg/L)	1.6	0–1.02
Lactate dehydrogenase (U/L)	1092.0	125–345
Lactate dehydrogenase isoenzyme 1 (U/L)	75.0	15–65
Creatine kinase (U/L)	200.0	25–180
Creatine kinase-MB (U/L)	78.8	0–25
N-terminal pro-B-type natriuretic peptide (pg/ml)	5632.0	0–125.2
High-sensitive cardiac troponin T (ng/ml)	0.418	0–0.014
Blood pH	7.27	7.35–7.45
Lactic acid (mmol/L)	5.5	0.5–2.22
Potassium (mmol/L)	3.85	3.9–5.4
Chloride (mmol/L)	110.50	98–110
Calcium (mmol/L)	2.03	2.1–2.8
Serum glucose (mmol/L)	11.60	3.89–6.11
Procalcitonin (ng/ml)	0.71	≤0.05
High-sensitivity C-reactive protein (mg/L)	21.40	0–3
Ferritin (ng/ml)	20269.98	12–135
Routine coagulation testing		
Activated partial thromboplastin time (s)	76.3	21.1–36.5
Prothrombin time (s)	23.4	9.7–12.6
D-dimer (mg/L FEU)	130.28	0–0.55
Fibrinogen (g/L)	1.53	1.92–4.01
Cytokine detection		
Interleukin-6 (pg/ml)	32367.87	0–20.9
Interleukin-10 (pg/ml)	354.37	0–5.9
Tumor necrosis factor α (pg/ml)	7.75	0–5.5
Interferon-γ (pg/ml)	22.01	0–17.3
Peripheral blood lymphocyte subsets		
Total T lymphocytes (%)	35.9	38.56–70.06
Natural killer cell (%)	6.63	7.92–33.99
Natural killer cell (/µl)	205	210–1514
CD19+ B lymphocytes (/µl)	54.79	10.86–28.03
CD19+ B lymphocytes (%)	1693	240–1317
Urine routine test		
Urine pH	5.0	5.5–7.5
Urine glucose	3+	–
Red blood cells	2+	–
Urinary albumin	+-	–

Chest radiographs indicated bronchopneumonia. Neurophysiological evaluation showed bilateral abnormalities in somatosensory evoked potentials (SEP): left-sided stimulation elicited only the N9 wave, with absent N13 and N20 waves; right-sided stimulation elicited N9 and N13 waves, but N20 was not detectable, suggesting central conduction dysfunction. Electroencephalography (EEG) showed generalized low-voltage activity, indicating severe cerebral suppression.

**Figure 1:**
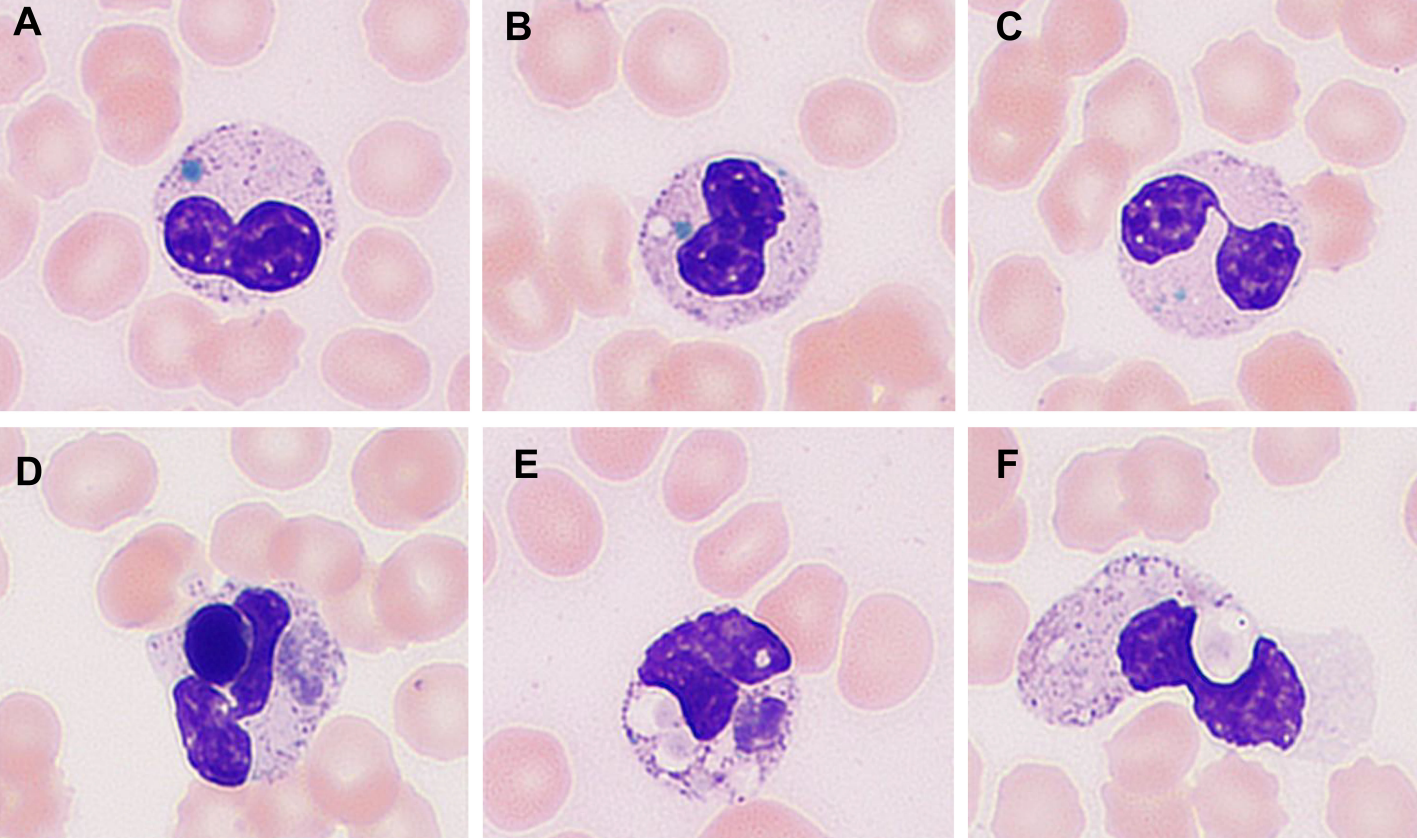
Peripheral blood smear findings (Wright–Giemsa staining). Panels **(A–C)** illustrate neutrophils containing distinctive blue-green cytoplasmic inclusions. Panels **(D–F)** highlight neutrophils exhibiting hemophagocytosis: panel **(D)** shows engulfment of a lymphocyte, while panels **(E, F)** demonstrate engulfment of erythrocytes.

**Figure 2:**
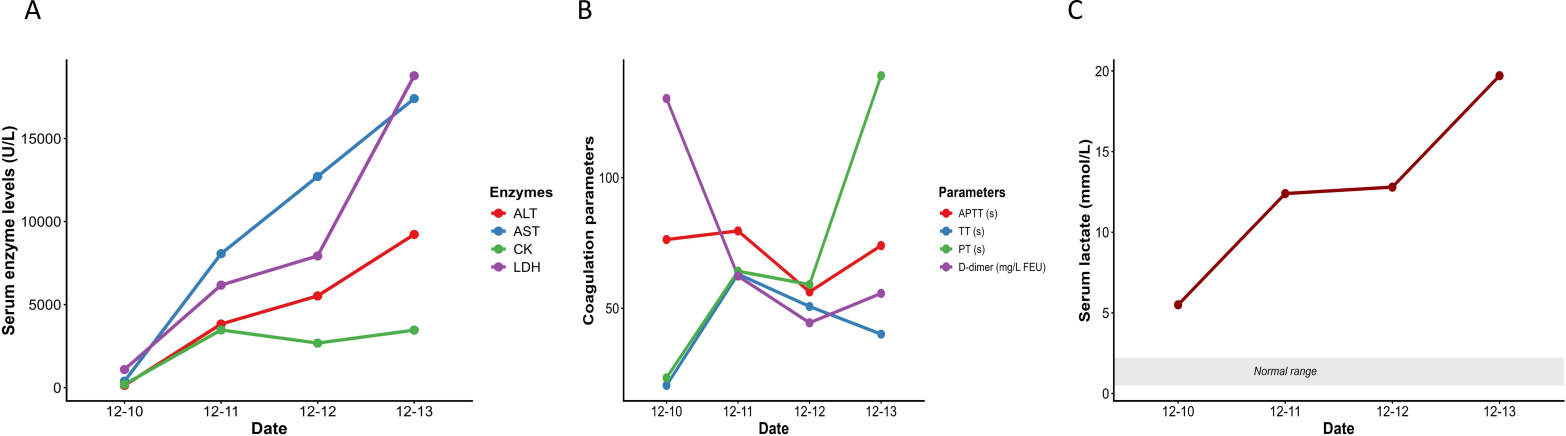
Dynamic trends of biochemical and coagulation markers over 4 days. **(A)** Serum enzyme levels (ALT, AST, CK, LDH; U/L) displayed marked elevation. **(B)** Coagulation parameters (APTT, TT, PT in seconds; D-dimer in mg/L FEU) demonstrated persistently abnormal values, with TT prolonged after admission. **(C)** Serum lactate (mmol/L) showed a progressive increase, exceeding the normal reference range.

On December 12, peripheral blood smear revealed blue-green inclusions in the cytoplasm of neutrophils. Additionally, neutrophilic phagocytosis of lymphocytes and erythrocytes was observed (**Figure 1**). During the 4-day hospitalization, the patient’s enzymatic markers showed a continuous and significant elevation: ALT increased from 132 U/L at admission to 9224 U/L, AST from 388 U/L to 17,406 U/L, LDH from 1092 U/L to 18,782 U/L, and CK from 200 U/L to 3462 U/L. Lactate (LAC) rose from 5.5 mmol/L to 19.71 mmol/L. Regarding coagulation parameters, thrombin time (TT) was within the normal range (20.5 seconds) at admission but became abnormal thereafter; APTT, PT, and D-dimer were elevated from the time of admission and remained persistently high throughout the course (see **Figure 2** for dynamic changes).

Microbiological studies on December 12 revealed that Gram staining of endotracheal aspirate showed Gram-positive cocci (4+) and Gram-negative bacilli (2+) with evidence of leukocyte phagocytosis. Imaging on December 12 showed bilateral pulmonary consolidation, bronchopneumonia, and small bilateral pleural effusions. Cardiac ultrasound revealed mildly reduced left ventricular systolic function and a small pericardial effusion.

## Treatment and clinical course

Comprehensive supportive care and multi-organ protection were initiated immediately upon admission. Respiratory support was provided via invasive mechanical ventilation. Hemodynamic stabilization included fluid resuscitation, correction of metabolic acidosis, and administration of multiple vasoactive agents (norepinephrine, epinephrine, dopamine, dobutamine). In terms of infection control, the patient was treated sequentially with broad-spectrum antibiotics, including ceftriaxone, meropenem, teicoplanin, and ceftazidime, in combination with antiviral agents such as oseltamivir, peramivir, and acyclovir. Immunomodulatory therapy included high-dose methylprednisolone, intravenous immunoglobulin, tocilizumab, and ulinastatin. Neurological protection involved the use of sedatives and analgesics to control seizures and minimize brain injury. For organ protection, targeted supportive therapies were administered for hepatic, renal, and cardiac function. In addition, the patient received blood product transfusions and anticoagulant therapy, along with extracorporeal blood purification through plasma exchange combined with continuous veno-venous hemodiafiltration (CVVHDF).

Despite aggressive multi-system interventions and advanced life support, the patient’s condition rapidly deteriorated. On hospital day 4 (December 13, 2024), continuous EEG monitoring demonstrated persistent electrical silence, and death was declared clinically.

## Discussion

This case describes a rare and rapidly fatal presentation of *influenza A virus* infection in a female toddler conceived via *in vitro* fertilization, who developed fulminant sepsis, ANE, and MODS. The child presented with abrupt onset of high fever, frequent seizures, altered consciousness, and respiratory failure, progressing swiftly to septic shock. Laboratory findings indicated a profound hyperinflammatory state characterized by elevated cytokines, coagulation abnormalities, liver and myocardial injury, and lactic acidosis. Neurophysiological studies, including continuous EEG suppression and abnormal somatosensory evoked potentials (SEP), strongly suggested the diagnosis of ANE—a rare but often devastating complication of viral infections in children. A particularly striking feature in this case was the identification of two rare hematological findings in the peripheral blood smear: blue-green neutrophilic inclusions (BGNIs) and neutrophilic phagocytosis of erythrocytes and lymphocytes. To our knowledge, this is the first pediatric case of influenza A infection demonstrating the simultaneous presence of both phenomena.

BGNIs, also colloquially referred to as “death bodies,” are rare cytoplasmic inclusions thought to contain lipopigments such as lipofuscin or biliverdin, possibly reflective of severe oxidative stress and hepatocellular injury. These inclusions have been predominantly described in critically ill adult patients with acute liver failure, lactic acidosis, or overwhelming infections and are frequently associated with poor short-term prognosis (Courville et al., 2017; Rivera et al., 2022). In this case, the presence of BGNIs was accompanied by markedly elevated liver enzymes (ALT, AST), lactate dehydrogenase (LDH), hyperferritinemia, and persistent lactic acidosis—findings that align with previous reports and support the association between BGNI formation and systemic metabolic derangement (Yang and Gabali, 2018). Notably, the persistent elevation of myocardial enzymes and small pericardial effusion in this case suggest concomitant myocardial injury, raising the possibility that BGNIs may indicate not only hepatic but broader multisystem oxidative and inflammatory damage.

In addition, neutrophilic phagocytosis of erythrocytes and lymphocytes—another unusual finding—was observed. This phenomenon is more typically associated with hyperinflammatory states such as hemophagocytic lymphohistiocytosis (HLH) and reflects excessive activation of the innate immune system (Canna and Marsh, 2020). The detection of hemophagocytosis in peripheral blood, rather than in bone marrow, may offer a valuable early clue for the presence of systemic immune dysregulation. In this patient, the constellation of laboratory findings—including extreme hyperferritinemia, elevated inflammatory cytokines (IL-6, IL-10, TNF-α, IFN-γ), and multi-organ involvement—supports the presence of a cytokine storm or secondary HLH phenotype triggered by influenza A infection (Fiore-Gartland et al., 2017).

Despite early and aggressive multidisciplinary treatment, including respiratory and circulatory support, broad-spectrum antimicrobials, immunomodulation, organ support, and blood purification (Chen et al., 2024), the patient’s condition deteriorated rapidly, culminating in MODS and DIC by day 4 of illness. This rapid course highlights the fulminant nature of viral sepsis in young children and raises questions about the roles of viral tropism, host immune response, and potential genetic susceptibility in determining disease severity.

This case underscores the rare occurrence of a fulminant and rapidly progressive course of influenza A H1N1 infection in a toddler. Although multiplex PCR screening for 13 common respiratory pathogens confirmed influenza A H1N1 (2009) RNA positivity and excluded other viral or atypical bacterial infections, metagenomic sequencing was not performed, which is a limitation of this report. Rare or unexpected pathogens beyond the detection range of targeted assays could not be entirely ruled out. Nevertheless, the clinical presentation, laboratory findings, and temporal progression strongly support H1N1 as the primary pathogen underlying the severe illness in this patient despite the absence of metagenomic sequencing.

In conclusion, this case illustrates two rare but potentially significant hematological features—BGNIs and neutrophilic hemophagocytosis—that may serve as morphological biomarkers of severe systemic inflammation in pediatric viral sepsis. Recognition of these features in peripheral blood smears could aid in early identification of high-risk patients and prompt escalation of care. Further studies in larger cohorts are needed to elucidate the pathophysiological mechanisms underlying these findings and to assess their utility in prognostication and clinical decision making.

## Data Availability

The original contributions presented in the study are included in the article/supplementary material. Further inquiries can be directed to the corresponding author.
